# Mosquito Feeding Affects Larval Behaviour and Development in a Moth

**DOI:** 10.1371/journal.pone.0025658

**Published:** 2011-10-03

**Authors:** Véronique Martel, Fredrik Schlyter, Rickard Ignell, Bill S. Hansson, Peter Anderson

**Affiliations:** 1 Department of Plant Protection Biology, Chemical Ecology, Swedish University of Agricultural Sciences, Alnarp, Sweden; 2 Department of Evolutionary Neuroethology, Max Planck Institute for Chemical Ecology, Jena, Germany; French National Centre for Scientific Research - Université Aix-Marseille, France

## Abstract

Organisms are attacked by different natural enemies present in their habitat. While enemies such as parasitoids and predators will kill their hosts/preys when they successfully attack them, enemies such as micropredators will not entirely consume their prey. However, they can still have important consequences on the performance and ecology of the prey, such as reduced growth, increased emigration, disease transmission.

In this paper, we investigated the impact of a terrestrial micropredator, the yellow fever mosquito *Aedes aegypti*, on its unusual invertebrate host, the Egyptian cotton leaf worm, *Spodoptera littoralis*. Larvae developing in presence of mosquitoes showed a slower development and reached a smaller pupal weight when compared to a control without mosquitoes, apparently because of a reduced feeding time for larvae. In addition, larvae tended to leave the plant in presence of mosquitoes.

These results suggest that mosquitoes act as micropredators and affects lepidopteran larvae behaviour and development. Ecological impacts such as higher risks of food depletion and longer exposure to natural enemies are likely to be costly consequences. The importance of this phenomenon in nature – the possible function as last resort when vertebrates are unavailable – and the evolutionary aspects are discussed.

## Introduction

Trophic strategies are separated into different categories based on the number of victims, distinguishing between parasites and predators, and the victim's fitness, dividing the predators into micropredators and predators [1]. Micropredators are natural enemies attacking more than one victim in their life, but not necessarily killing it, such as leeches, lampreys or ticks, and are sometimes considered as mobile temporary parasites [1]–[3]. A micropredator is usually smaller than its prey, and the size of the meal is then constrained by the micropredator size, and not that of the prey [2].

Most studies on micropredators concern vertebrate prey in aquatic systems, such as isopods or dinoflagellates feeding on fish [3]–[5], or terrestrial systems including mosquitoes, ticks and flees and their vertebrate prey [6], [7]. Just like predators or parasitoids can affect their prey/host without attacking them - non-consumptive effects or traits-mediated interactions - micropredators can also affect the performance of their prey without killing them. For example, micropredators can reduce their prey's growth rate [7], modify their competitive ability [8], and alter swimming behaviour of fish [3].

Female mosquitoes are blood-feeding micropredators that are attracted to and feed on a wide variety of vertebrates, from amphibians to mammals, in order for their eggs to develop [9], [10]. However, it has been reported punctually in the literature that blood-feeding insects occasionally feed on invertebrates, such as insects. In the oldest report, Hagen [11] reported black flies (*Simulium*) feeding on butterfly pupae. Cicadas and small dipterans have also been observed being attacked by mosquitoes [12]. Similarly, Waage [13] reported that some biting midges of the genus *Forcipomyia* can feed on insect haemolymph. Such a behaviour has been described in more details in the laboratory using *Aedes aegypti* and *Culex tarsalis* [14], [15]. The authors found that these mosquitoes were attracted to Lepidoptera larvae and were feeding on them, and that egg production occurred following a haemolymph meal [15].

Haemolymph feeding by blood-feeding insects brings many questions on ecology and evolution of this phenomenon. Although it has been shown that mosquitoes suffer a cost from haemolymph feeding through an inferior egg production [14], nothing is known on the impact for the insects serving as prey. Do lepidopteran larvae respond to the presence of mosquitoes and can micropredation by mosquitoes affect larval behavior and their fitness?

In this paper, we investigated the impact of a micropredator in a terrestrial system, the yellow fever mosquito *Aedes aegypti* (Diptera: Culicidae), on the performance and behaviour of an unusual invertebrate prey, the Egyptian cotton leaf worm *Spodoptera littoralis* (Lepidoptera: Noctuidae). These two species co-occur in natural systems, for example in Egypt. Experiments were conducted to test if the mosquito attacks actually result in a haemolymph meal for the female mosquitoes, and to investigate if larvae suffer from any costs consequently to these attacks. Although death is not expected to occur following micropredation, we hypothesize that larvae in the presence of mosquitoes, their micropredators, should suffer physiological costs e.g. extended developmental time and reduced growth. Furthermore, we tested if presence of micropredators affects larval behaviour by investigating propensity to migrate/move away from their feeding plant. To our knowledge, this the first study on the impact of a micropredator on an invertebrate prey.

## Materials and Methods

### Insects

One to two weeks old, non-blood fed male and female *Aedes aegypti* of the Rockefeller strain obtained from the Liverpool School of Tropical Medicine, replenished with new mosquitoes from Liverpool School of Tropical Medicine in 2007, were used in our experiments. Larvae were reared in plastic containers (20×1×7 cm) and fed with Tetramin fish food. Pupae were put in a small plastic cup and transferred to plastic cages (size) under 27°C, 70–80% r.h. and a 12∶12 h LD photoperiod. Adults had access to 10% sugar solution presented on a filter paper.

*Spodoptera littoralis* used in this study originated from a laboratory culture established in 2007 with moths from Egypt, reared on an artificial diet [16] using potatoes instead of beans. Wild-collected moths from Egypt have been introduced into the culture at least once annually since the start of the culture. All stages of the moths were kept at 25°C, 70% r.h. and LD 16∶8 h.

### Plants

Cotton plants, *Gossypium hirsutum* (L.) (Malvaceae, var. Delta Pineland 90) were kept in a climatized greenhouse at 25±5°C, 70±10% r.h.. Artificial light (Osram Powerstar, HQI-T, 400 W/D, Daylight) was provided in addition to natural light from October until April. The plants were individually grown from seeds in 14 cm diameter pots. Cotton plants were used when they reached 8–10 fully developed true leaves. No flowering plants were used.

### Mosquito attacks and feeding

To assess if both male and female mosquitoes were attracted to *S. littoralis* larvae, 2 early 6^th^ instar larvae were introduced in a 1 liter container together with 10 males (n = 5) or 10 females (n = 8) for 20 minutes. The number of landings was observed.

To assess if female mosquitoes ingest haemolymph from *S. littoralis* larvae when they landed, 3^rd^ instar larvae were fed using artificial food (described above) mixed with the colour pigment Xylene cyanole FF until the food medium became strongly blue. When they reached the late 5^th^ instar, two larvae were placed in a 1 liter container together with about ten 10–12 days old female *A. aegypti*, non-blood fed but sugar-fed (n = 8). After 48 h, the mosquitoes were dissected in order to assess the presence of the blue dye in their digestive tract, indicating a haemolymph meal.

### Larval feeding

To evaluate the impact of mosquitoes on the time invested in feeding in *S. littoralis* larvae, one potted cotton plant was placed in a Nylon and plastic cage (47.5×47.5×93.0 cm, BugDorm-4180F, Megaview Science Co, Ltd, Taiwan). In one cage, 40–50 *A. aegypti* adults (males and females) were introduced while in a control cage, no mosquitoes were introduced. One 4^th^ instar *S. littoralis* larva, starved for 12–18 h, was introduced in the cage. Before introduction, the larvae were presented with one cotton leaf and only the larvae that started feeding were selected. The observation started as soon as the larva uncurled and finished after 20 minutes (n = 14 of each treatment). During that period, the time spent feeding and the number of mosquitoes landing on the larva for the treatment with *A. aegypti* was recorded using a homemade software, ObserverPi (Lund University). The observations in which the larva did not feed were removed (one in each treatment). All experiments were performed in a greenhouse where temperature varied between 20 and 30°C, and r.h. between 40% and 70%, under natural light.

### Larval migration

To evaluate the impact of mosquitoes on the tendency of the larvae to leave a plant, one potted cotton plant was placed in the middle of a 120×80×60 cm mesh cage covered with gauze. Paper covered with Tangle trap adhesive (The Tanglefoot Company, Grand Rapids, Michigan, USA) was then placed around the pot, at the level of the border of the pot, in order to catch any larva moving away from the plant. In one of the cages, 40–50 *A. aegypti* adults (males and females) were introduced twice a week while in a control cage, no mosquitoes were introduced. Multiple introductions of mosquitoes were necessary as mosquitoes also tended to get caught on the adhesive paper. Sucrose solution was introduced in the cage for a few hours every 3–4 days to prevent mosquitoes from dying from starvation. Five 4^th^ instar larvae were introduced on the plant. After one week, the number of larvae trapped on the adhesive paper was counted. All experiments (n = 6 of each treatment) were performed in a greenhouse where temperature varied between 20 and 30°C, and r.h. between 40% and 70%, under natural light.

### Larval performance

To study the effect of mosquitoes on *S. littoralis* larval performance, one potted cotton plant with five 12 days old *S. littoralis* larvae (3^rd^ instar) of similar mass were introduced into a Nylon and plastic cage (47.5×47.5×93.0 cm, BugDorm-4180F, Megaview Science Co, Ltd, Taiwan). The top of the pots were covered with aluminum foil and the holes in the bottom were closed with tape to prevent larvae from pupating in the soil. In half of the cages, 40–50 *A. aegypti* adults (males and females) were introduced, the remaining cages serving as control. The larvae were weighed before being introduced in the cage (age 12 days), and when 16 days and 20 days old. The cages were checked for pupae every day until all larvae had pupated. Development time was noted, and pupae were sexed and weighed as soon as fully sclerotized. Sucrose solution was introduced in the cage for a few hours every 3–4 days to prevent the mosquitoes from dying from starvation. All experiments (n = 11 of each treatment) were performed in a greenhouse where temperature varied between 20 and 30°C, and r.h. between 40% and 70%, with both natural and artificial lights from October until April.

### Statistical Analysis

All statistical analyses were performed using GraphPad 5.01 (GraphPad Prism version 5.01 for Windows, GraphPad Software, San Diego California USA, www.graphpad.com). For the larval feeding experiment, a non-parametric Mann-Whitney test was conducted to compare the time spent feeding by larvae in the control group and in the mosquito group.

For the larval migration experiment, a non-parametric Mann-Whitney test was conducted to compare the number of larvae that left the plant (and were trapped on the adhesive paper) in each cage in the control group and in the mosquito group.

For the larval performance experiment, all statistical tests were done on the average values from the five larvae (or the surviving ones) in each cage. A two-way ANOVA with repeated measures was performed to compare larval weights depending on the presence of mosquitoes. Pupal weights, development time and number of surviving larvae in the control and in the mosquito groups were compared using a *t*-test. To compare sex ratio between the two groups, a 2×2 contingency table was done and a χ^2^ was calculated.

The effect sizes of treatments were also calculated for all variables measured [17]. The ‘effect size’ is a unit less measure of the strength of the relationship between two variables obtained by dividing the difference between two means with the pooled standard deviation for those means, following the equation:
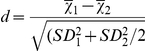



Following Cohen's criteria [18], |d| = 0.2 indicates a small effect, |d| = 0.5 indicate a medium effect and |d| = 0.8 indicates a strong effect.

## Results

### Mosquito attacks and feeding

**Figure 1:**
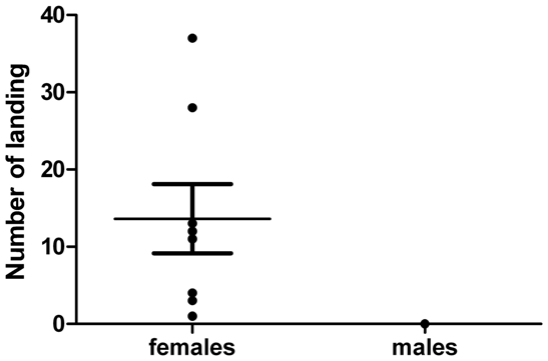
*Aedes aegypti* females landing on *Spodoptera littoralis* larvae. Mean (±SE) number of landing for male (n = 5) and female (n = 8) *Aedes aegypti* on early 6^th^ instar *Spodoptera littoralis* larvae during a 20 min observation period.

No males were observed landing on the larvae, while on average (±SE) 13.6±4.5 female landings were observed during the 20 min observation period (Figure 1). The landings mostly occurred on the posterior end of the larvae and larvae generally tried to dislodge the mosquito by moving their head backwards towards the landed mosquito (Video S1).

Ten out of the 68 mosquito females dissected (14.7%) contained blue dye in their digestive tract after spending 48 h with the dye-fed larvae, indicating the presence of haemolymph.

### Larval feeding

Larvae in the control cages spent significantly more time feeding than in the mosquito treatment (3125±367 s vs. 2002±379 s; U = 44.0, d.f. = 1, *P* = 0.014). The effect size (Table 1), d = −11.3, must be considered as very large. In the mosquito cages, an average (±SE) of 31±14 mosquito landings were observed by 20 min period, for a total of 465 landings observed overall.

**Table 1:** Effect sizes of the impact of the presence of *Aedes aegypti* on the larval performance and behaviour of *Spodoptera littoralis* growing on cotton plants.

Variable measured	Effect size (d)
Feeding time	−11.3
Emigration	2.24
Development time	1.17
Weight	
12 days	0.15
16 days	−0.30
20 days	−0.52
Pupae	−1.03
Survival	−0.15
Sex ratio	0.05

An absolute value of 0.2 corresponds to a small effect, 0.5 to a medium effect and 0.8 to a large effect [19].

### Larval migration

**Figure 2:**
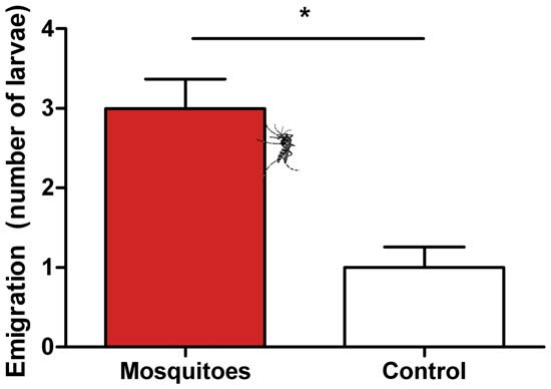
Emigration rate of *Spodoptera littoralis* larvae in presence or absence of mosquitoes. Number of *Spodoptera littoralis* larvae that got trapped in the adhesive paper after one week, when migrating from cotton plants in the presence or absence of the mosquito *Aedes aegypti* (n = 6 of each treatment). Mann-Whitney test: U = 1.00, d.f. =  1, *P* = 0.0065.

More larvae were caught on the adhesive paper in the cage containing mosquitoes than in the control cage (U = 1.00, d.f. = 1, *P* = 0.0065) (Figure 2).

### Larval performance

**Figure 3:**
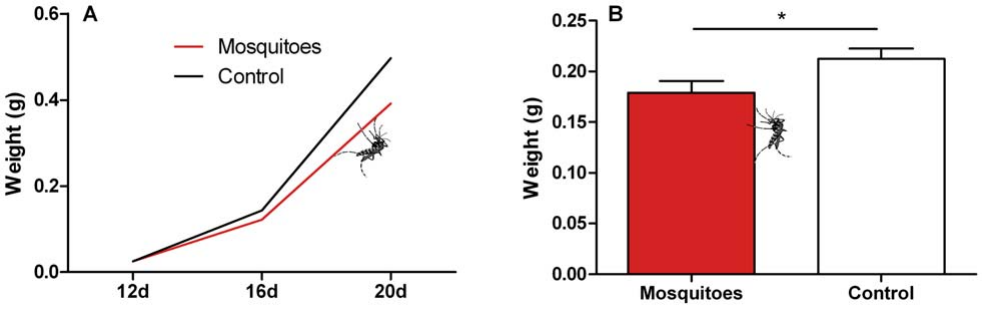
Weight of *Spodoptera littoralis* growing in presence or absence of the mosquito *Aedes aegypti*. Larval weight (mean ± SE, n = 11). Two-way ANOVA with repeated measures: Age: F = 116.8, d.f. = 2, *P*<0.0001; Presence of mosquitoes: F = 1.66, d.f. = 1, *P* = 0.22. **B.** Pupal weight (mean ± SE, n = 11). T-test, t = 2.18, d.f. = 20, P = 0.041.

Larval weight increased with age (F = 116.8, d.f. = 2, *P*<0.0001), as expected, but was not influenced by the presence of mosquitoes (F = 1.66, d.f. = 1, *P* = 0.22) (Figure 3A). However, pupal weight was significantly higher in control treatment compared to mosquito cages (Figure 3B, t = 2.18, d.f. = 20, P = 0.041). In addition, mosquito presence had a medium and large effect size on larval weight at 20 days and pupal weight, respectively (Table 1; d = −0.52 and −1.03).

**Figure 4:**
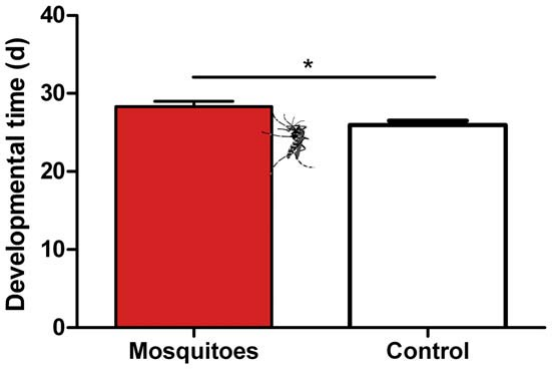
Development time of *Spodoptera littoralis.* Time from hatching to pupation for *S. littoralis* larvae growing on cotton plants in presence or absence of the mosquito *Aedes aegypti* (n = 11 for each treatment). T-test, t = 2.71, d.f. = 20, *P* = 0.014.

Larvae in the presence of mosquitoes had a significantly higher developmental time than larvae in control cages (Figure 4, 33.0 days against 28.5 days; t = 2.71, d.f. = 20, *P* = 0.014) and the resulting effect size was large (Table 1; d = 1.170). There was no difference in survival (t = 0.35, d.f. = 20, *P* = 0.73) or sex ratios (χ^2^ = 0.15, d.f. = 1, *P* = 0.69) between the two treatments.

## Discussion

Female *A. aegypti* are attracted to, land on and sometimes feed on *S. littoralis* larvae. In response to these attacks, the larvae try to dislodge them by moving their head, clearly showing that larvae perceived the mosquitoes. This response is similar to that to parasitoids [19]. The larval behaviour probably explains the low mosquito feeding success rate, as it depends on the micropredator not being detected by its prey [20]. Indeed, defensive behaviour is common in vertebrate prey and is known to affect the foraging success of mosquitoes [21]. In addition, the success rate of feeding is generally positively correlated with prey size for vertebrates, with smaller animals having more intensive and efficient defensive behaviour [21]. Although the success rate of feeding is rather small for mosquitoes (14.7%), we did observe an actual cost of the presence of mosquitoes during larval development. We found a reduced energy income (through less feeding), lower pupal weight and increased emigration in the present study.

Larvae emigrated more in presence of mosquitoes, again indicating that larvae perceived the mosquitoes as a potential danger, or at least as a disturbance. The migration to another plant can delay the development because of time and energy spent migrating instead of feeding, but also increase the mortality risk [22], and a risk of not finding any adequate host plant in the surroundings [23].

When no migration was possible, the larvae showed longer developmental time and smaller pupae in presence of mosquitoes, probably caused by a reduction in feeding time. A reduced feeding time has also been observed in vertebrate prey such as cattle [24]. Such an increase in the development time can have significant impact on insects.

Having a longer development time also translate in a longer window frame during which the larvae are susceptible to attacks from their more well-known natural enemies like predators and parasitoids (slow-growth-high-mortality hypothesis, [25], [26]). The mortality risks would be higher for larvae on which mosquitoes are feeding than for other larvae. Such higher mortality risks indirectly caused by micropredators have also been shown in fish, where erratic behaviour is expected to attract predators [3].

Pupae reached a larger size when no mosquitoes were present during the larval stage. Reduced growth caused by micropredation is also known in marine systems [8]. A larger pupa translates into a larger adult, and adult size is known to affect fitness in insects: large females have a longer longevity [27], [28] and a higher fecundity (reviewed by Honek [29]). It is then likely that larvae attacked by mosquitoes will produce adults with a lower fitness. Reduced performance caused by mosquitoes has also been observed in vertebrate preys [21].

Finally, even if not measured in this study, additional potential costs can be incurred by mosquitoes' attacks. Mosquitoes inject saliva when they feed [30] and this can initiate an immune response against this foreign compound in the larvae. It is known that immune response is costly in insects, inducing a higher metabolic rate, a lower survival rate, a lower tolerance for desiccation and starvation, a decreased fecundity and a decreased life span among others [31], [32]. Another possible impact for the larvae could be disease transmission, which is common for micropredators [20]. Insect larvae can be infected by numerous pathogens and there is then a risk of transmission if a mosquito female feeds on two different larvae. Parasitoids can transfer diseases and bio-insecticides such as *Bacillus thuringiensis* (reviewed by Quicke [33]).

The importance of this phenomenon in nature remains to reveal, as discussed by Harris et al. [15] and is outside the scope of this study. Are these acts of mosquito micropredation incidental under specific lab or field conditions, or a consequence of an evolutionary past of insect feeding?

It has been suggested earlier [15] that invertebrate preys, even if not the preferred ones, might be used in last resort: either at the end of the life of a mosquito, or when vertebrates are scarce. It is also possible that when mosquitoes look for a shelter in windy environment, they often end up in habitats where they encounter insect larvae. That could be a situation where feeding on insect larvae in proximity might be an alternative to leaving the shelter to find vertebrate hosts, but being exposed to adverse conditions, such as wind and rain [34].

An alternate hypothesis is that this attraction might be a remnant of their ancestors feeding behaviour. Haematophagy in mosquitoes could either have evolved from plant feeding (feeding on plant sap or piercing fruits) or be derived from predation on other insects (reviewed in Waage [13] and Balashov [35]). In either case, mosquitoes could have gone through a stage of micropredation, feeding on insect haemolymph before developing the ability to suck blood from vertebrate prey. Without any selective pressure against the attraction to insect prey, they could still possess this attraction even though it normally would not occur if vertebrate prey is available. Further studies are warranted to investigate this hypothesis.

## Supporting Information

Video S1 **A female** ***Aedes aegypti*** **repeatedly landing on a 6^th^ instar** ***Spodoptera littoralis*** **larva.** The larva is moving its head towards the landed mosquito to get rid of it.
(MP4)
